# Selected Potential Biomarkers in Laryngeal Cell Carcinomas

**DOI:** 10.3390/cancers18030477

**Published:** 2026-01-31

**Authors:** Roman Paduch, Maria Klatka, Janusz Klatka

**Affiliations:** 1 Department of Virology and Immunology, Faculty of Biology and Biotechnology, Maria Curie-Skłodowska University, 20-033 Lublin, Poland; 2 Chair and Department of General and Paediatric Ophthalmology, Medical University of Lublin, 20-079 Lublin, Poland; 3 Department of Paediatric Endocrinology and Diabetology, Medical University, 20-093 Lublin, Poland; maria.klatka@umlub.edu.pl; 4 Department of Otolaryngology and Laryngological Oncology, Medical University of Lublin, 20-954 Lublin, Poland; janusz.klatka@umlub.edu.pl

**Keywords:** biomarkers, laryngeal cell carcinomas, cancer detection, prediction

## Abstract

Laryngeal squamous carcinoma is a major type of head and neck cancer. Despite a wide range of treatment options, it remains a challenge to identify which ones are the most effective for which groups of patients. One solution is to analyse selected biomarkers. In this paper, biomarkers are divided into distinctive groups according to the molecular pathways analysed or specific molecules within the cell or in tissue fluids. The paper provides a description of these groups, including genetic and apoptosis-associated factors, factors regulating angiogenesis, cell structure regulators, immune factors in the form of programmed cell death ligand (PD-L1), hormone receptors, molecules involved in growth factor pathways, and cell cycle regulators. Representative examples are discussed for each of these groups, indicating their potential usefulness in staging, assessing tumour aggressiveness, and making a prognosis.

## Simple Summary

Diagnosing head and neck cancers and predicting the aggressiveness and metastatic potential of these tumours currently constitutes a significant therapeutic challenge. Analysing tumour markers associated with and specific to various types and locations of laryngeal carcinomas is therefore an approach that allows for rapid and effective diagnosis. The aim of this paper is to present selected molecular factors that may be considered useful in the diagnosis of laryngeal carcinomas. We believe that their use in clinical practice may, in many cases, have a positive impact on specifying or justifying the diagnosis and predicting the aggressiveness of detected neoplastic lesions.

## 1. Introduction

Laryngeal squamous carcinoma is currently the second most common head and neck cancer. For clinical purposes, laryngeal carcinomas have been divided into those located in the supraglottic, glottic, and subglottic regions [1]. Both dysplasia, i.e., a lesion not extending beyond the basement membrane, and carcinoma in situ of the laryngeal mucosa can develop into laryngeal cancer. However, instead of remaining dormant in situ, this cancer may directly develop into an invasive type [1,2]. The most common causes of this cancer are smoking and alcohol abuse. Other notable factors include inhaled toxic agents present in coal, cement, and wood dust, as well as certain chemicals such as polycyclic aromatic hydrocarbons, benzopyrene, aflatoxin, heavy metals (cadmium, lead, caesium), radiation, and viruses such as Epstein–Barr virus (EBV) or oncogenic types of human papillomavirus [3,4,5,6]. Squamous cell carcinoma (SCC) is the most prevalent laryngeal tumour, accounting for over 95% of diagnosed cases. Treatment for SCC can be divided into surgical and non-surgical. The former includes surgical treatments based on partial or total laryngectomies. The latter includes radiation therapy (RT), chemoradiotherapy, and in some cases, immunotherapy, which appears to be effective [4]. Biomarker analysis of laryngeal cancer tissue or cells isolated from it is used to identify which methods will provide the best outcomes for individual groups of patients

Biomarkers that can be used in the diagnosis of squamous laryngeal carcinoma can be divided into distinctive groups depending on the analysed molecular pathways or specific molecules within the cell or in tissue fluids [7]. Table 1 lists selected molecules described in this paper as potential biomarkers in laryngeal cancer.

## 2. Genetic and Apoptosis-Associated Factors

### 2.1. Long Non-Coding RNAs (LncRNAs)

LncRNAs constitute a class of non-coding RNAs (ncRNAs) with transcripts exceeding 200 nucleotides in length. They participate in the regulation of gene transcription, which influences numerous cellular processes, including proliferation, mobility, apoptosis, and angiogenesis, as well as epigenetic phenomena in the cell, such as histone methylation. Many lncRNAs—often constituting oncogenes or tumour suppressor genes—are aberrantly expressed in tumours, which significantly affects their phenotype. Therefore, they can be classified as molecular markers, and by identifying a characteristic pattern, they can be considered biomarkers of specific tumours. In the case of squamous laryngeal carcinoma, numerous aberrantly expressed lncRNAs have been found [8]. Examples include AFAP1-AS1 (LncRNA actin filament-associated protein 1 antisense RNA1) and HOTAIR (Homeobox transcript antisense RNA), which are both diagnostic and prognostic markers in nasopharyngeal cancer (NPC). HNF1A-AS (Hepatocyte nuclear factor 1A antisense RNA) and LncRNA-ROR are, in turn, molecular therapeutic targets in the same tumour [9]. Other examples of lncRNAs associated with head and neck cancer include the following: AC104041, which stabilises the Wnt2B ligand and activates β-catenin, enabling head and neck cancer metastasis; LINC00680, which promotes the progression of oesophageal squamous cell carcinoma; PVT1, an oncogene regulating the proliferation of nasopharyngeal carcinoma cells and their resistance to apoptosis; and ANRIL, which promotes the proliferation and invasion of laryngeal squamous cell carcinoma [10]. Furthermore, selected mitochondrial-related drug-resistant lncRNAs can predict the prognosis and immune response of laryngeal squamous cell carcinoma (LSCC) and distinguish high-risk from low-risk patients. These types of lncRNAs could be considered as demonstrating prognostic significance and serving as biomarkers of the sensitivity of LSCC tumours to chemotherapy [11]. Hence, abnormal lncRNA expression can play a significant role in promoting or inhibiting the development of laryngeal cancer. Unfortunately, their prognostic value in assessing the risk of recurrence is fairly limited. However, the advantage of lncRNAs is often their specificity as biomarkers for certain tissues (tissue-specific) and entire tumours (cancer-specific), including laryngeal SCC. Moreover, they are stable in body fluids, and their detection requires non-invasive sampling methods and sensitive molecular and genetic biology techniques. Therefore, abnormal lncRNA expression plays a significant role in promoting or inhibiting the development of laryngeal cancer. Unfortunately, their prognostic potential for assessing the risk of recurrence is rather limited [8].

LncRNAs are also located in exosomes. Exosomes are a type of extracellular vesicle (EV) with a diameter of 40–160 nm, commonly found in various bodily fluids such as urine, blood, saliva, and cerebrospinal fluid. They are considered one of the mechanisms of intercellular communication. Exosomes are involved in the regulation of both physiological and pathological cellular phenomena. This stems from the fact that they contain numerous biologically active factors such as proteins, lipids, nucleic acids, and metabolites. In addition to lncRNAs, exosomes also contain microRNAs (miRNAs) and circular RNAs (circRNAs). It has been suggested that the genetic material located in exosomes may play a role in the development of cancer, primarily through the transfer of tumour genetic material and the induction of neoplastic transformation. Furthermore, the dysregulation of exosomal lncRNAs has been shown to influence treatment resistance, vascularisation, and metastasis in laryngeal cancers. For example, the lncRNA ADAMTS9-AS2 located in exosomes has been shown to influence the development and dissemination of oral squamous cell carcinoma cells (OSCC) [12]. Exosomal miRNA has also been proposed as a useful marker for LSCC. This is because this type of genetic material is abundant in the early stages of the disease and is non-invasively accessible from saliva [13]. Due to the stability of exosomes, they can be isolated from body fluids during liquid biopsy and used as material for early cancer diagnostic analyses [12,14].

In the context of laryngeal carcinoma, patients have been demonstrated to exhibit higher levels of protein-containing exosomes compared to healthy individuals. This makes it possible to distinguish patients with head and neck squamous cell carcinoma (HNSCC) between different stages of the disease (I/II and III/IV) [12]. Insulin-like growth factor binding protein 7 (IGFBP7) as well as annexin A1 (ANXA1) have also been implicated as potential exosomal biomarkers for the detection of LSCC [14].

In the context of analysing the genetic material of laryngeal cancer cells, it is also important to note changes in DNA content in cells, as estimated by the adjusted DNA index (aDI). Increased DNA levels in these cancer cells have been shown to correlate with shorter survival times due to a higher number of affected lymph nodes and thus an overall poorer prognosis. This index, i.e., increased DNA ploidy in a cell, may therefore also serve as a specific marker in the context of laryngeal cancer [15].

### 2.2. Proteins Regulating Apoptosis

These proteins may also serve as markers of laryngeal cancer progression. One example is the membrane protein Bcl-2. It blocks apoptosis by influencing the release of cytochrome c from mitochondria and modulating the activation of the caspase pathway. This protein has been shown to be clinically insignificant in terms of the prognosis, histopathological changes, and survival of patients with laryngeal cancer [16,17,18]. On the other hand, it has also been reported that Bcl-2 is modulated in laryngeal cancers, showing correlations with lymph node metastasis, location, and the likelihood of disease recurrence [7]. Furthermore, Nix et al. indicated that Bcl-2 may be associated with radioresistance in laryngeal cancer [19]. The p53 protein also plays an important role in the proapoptotic pathway. However, the analysis of this factor in laryngeal cancer is challenging due to differing interpretations of study results. This may be due to the different functions of mutated forms of this protein depending on the mutation site. Hence, the assessment of genetic abnormalities is recommended to clearly classify modifications of this protein and recognise it as a reliable predictor in patients with laryngeal cancer [20,21]. Nevertheless, Bradford et al. demonstrated the important role of p53 in a group of patients with advanced laryngeal carcinoma. Increased levels of this protein were associated with decreased survival in these patients [22]. Similarly, poor laryngeal carcinoma patient outcomes and decreased survival were demonstrated by Nylander et al. in the context of p53 mutations [22,23]. Nevertheless, co-expression of p53/bcl-2 in laryngeal cancer may be an interesting alternative, as it shows a higher predictive and prognostic value than the assessment of both parameters separately [24].

## 3. Cell Cycle Regulators

An important group of factors that can be categorised as markers in laryngeal carcinoma are cell cycle regulators. Some of the most frequently mentioned are cyclin D1, p16, and p27 proteins, as well as the nuclear proteins Ki-67 and proliferating cell nuclear antigen (PCNA).

### 3.1. Cyclin D1

The cell cycle must be strictly controlled in order to proceed properly. Cyclins, among others, are involved in this process. A key one is cyclin D1, considered a protooncogene, which not only controls cell cycle progression but is also a mitotic factor signal-responsive protein. Consequently, cyclin D1 forms complexes with the cyclin-dependent kinases CDK4 and CDK6, activating them, leading to the phosphorylation of the retinoblastoma protein (RB). This is followed by the dissociation of E2F transcription factors from the pRB complex, with the cell cycle transitioning from the G1 to S (synthesis) phase. The inhibition of cyclin D1 activity inhibits the cell division cycle, but the overexpression of cyclin D1 stimulates cell entry into the G1 and then S interphase, driving uncontrolled cell division and promoting carcinogenesis. Cyclin D1 expression levels may hence serve as an indicator (marker) of aggressive tumour growth, as well as of the malignant phenotype of tumour cells [25]. Mutations within the CCND1 gene encoding Cyclin D1 have been reported fairly commonly in laryngeal tumours. This causes gene alterations, followed by changes in the expression of this protein. This can lead to the dysregulation of the cell cycle, particularly in the transition from the G1 to S phase, deregulation of cell proliferation, and consequently development of laryngeal cancer. Gene mutations often lead to abnormal or inactive forms of proteins. This may also involve the degradation of the misfolded protein and the inhibition of its potential oncogenic functions. Nevertheless, data on the potential usefulness of this protein as a marker for the disease in question have been inconsistent [26,27]. It is considered possible to link the expression of this cyclin to the histological degree of malignancy of laryngeal cancer. However, it has also been noted that the level of this cyclin cannot be clearly indicated as an independent prognostic factor. Diagnostic performance could be improved by linking cyclin D1 expression with pRB protein levels [28]. The stage of the tumour and, by extension, its metabolic state are also significant factors here. In early stages, levels of this cyclin may be useful for differentiating laryngeal carcinoma (LC) from premalignant laryngeal lesions (PLL) and healthy laryngeal tissue. Thus, it can indicate and identify the degree of risk of developing head and neck cancer. In advanced stages, it is believed that Cyclin D1 overexpression may serve as a predictive marker of overall and disease-free survival, as well as of lymph node metastasis [29,30]. Furthermore, this protein may represent a therapeutic target, with its reduced expression potentially helping patients with LSCC. Other parameters, such as gender, patient age, geographical region, and even smoking history, do not show a correlation with changes in cyclin D1 levels and laryngeal cancer [30].

### 3.2. p16 Protein

The p16 tumour suppressor gene plays an interesting role in the pathogenesis and diagnosis of head and neck cancers. It encodes distinct transcript variant proteins that function as inhibitors of cyclin/CDK systems and negatively regulate CDKs 4/6, inhibiting phosphorylation of the Rb protein. This gene is situated in the INK4a/ARF locus and encodes three transcript variants: p14ARF, p12, and p16α. These variants are involved in suppressing tumour development and progression. This protein localises to the cytoplasm and nucleus, where it exerts its influence [5,31]. It has been suggested that high cytoplasmic p16 expression with concomitant low nuclear expression may be associated with poor prognosis for head and neck cancer patients. The p16 gene is inactivated in at least 50% of cancer patients. Reduced p16 gene expression and activity are currently associated with a more advanced stage of disease progression and a high likelihood of cancer cell dissemination throughout the body [32]. The functionality of p16 is based on the hypophosphorylation of Rb proteins, which bind to the E2F1 transcription factor and ultimately inhibit S-phase genes, leading to cell cycle arrest [5,32]. Changes in p16 expression may, therefore, be an early symptom of preneoplastic lesions, as well as a marker of advanced tumour progression. This may be associated with hyperphosphorylation of pRb protein and activation of uncontrolled cellular proliferation. Increased p16 expression has a fairly strong correlation with human papillomavirus (HPV)-related cancers. It has been shown that in HPV-related HNSCC, p16 is overexpressed, whereas in non-HPV-related HNSCC, this gene is inactivated, and the amount of p16 protein decreases. This phenomenon (p16 positivity) is used as a marker for HPV-positive tumours, demonstrating significant diagnostic sensitivity [31,33]. Hence, the p16 protein is an important prognostic marker in head and neck tumours of the oropharynx (HNSCC). High levels of p16INK4a expression have also been suggested as a potential marker of EBV infection in pharyngeal and nasopharyngeal tumours. Unfortunately, studies indicate that this protein has limited effectiveness as a reliable prognostic marker of progression in EBV-positive laryngeal cancers (LSCC) [5]. In summary, p16 has been shown to carry a similar diagnostic value as a marker for nonoropharyngeal, especially laryngeal, hypopharyngeal, and oral cavity cancers, as well as for oropharyngeal HNSCC cancers, but not for LSCC [34].

### 3.3. p27 Protein

Another gene and its product with potential marker status is p27. Like p16, this protein is a cyclin-dependent kinase inhibitor. However, it belongs to the Cip/Kip group of cyclin A/E-cdk2 inhibitors, resulting in cell cycle arrest in the G1 phase (G1/S transition of the cycle). In normal cells, this protein is expressed in the nucleus, whereas in cancer cells, there is a significant decrease or loss of p27 expression. The activity of p27 is associated with a reduction or inhibition of cell cycle progression, enabling the repair of DNA damage. However, when levels of this protein are significantly reduced, damage to genetic material is replicated and cell instability increases. This may also involve modification of the extracellular matrix and adhesion potential of the tumour microenvironment, causing an increase in the mobility of transformed cells. This, in turn, is associated with tumour progression, poor prognosis, and reduced patient survival. Such observations have been made in the case of squamous cell carcinoma of the head and neck, with decreased levels of p27 serving as a convenient marker of progression and an indicator of the stage [35]. In head and neck cancers, low expression of the p27 protein may, therefore, be an independent prognostic factor correlating with clinical aggressiveness, unfavourable course of possible treatment, and thus poor prognosis [36]. Furthermore, it has been suggested that this protein may also serve as a predictive marker of response to chemotherapy in HNSCC patients. This may be attributable to its role in regulating the cell’s response to DNA damage, mediating apoptosis-related effects and, by extension, the chemosensitivity of the cell [37]. This marker may also be a predictive factor in relation to sex, lymph node metastasis, and disease stage. What is more, it may correlate with p53 protein expression [38]. Moreover, in oral cancers, a decrease in p27/kip1 levels may already be found at the stage of advanced dysplasia, suggesting that this marker can play a role in the early stages of the disease. Hence, the p27 protein appears to be an independent predictive and prognostic marker in head and neck cancers [36].

Among cell cycle regulators, Ki67 and PCNA proteins are also important markers of head and neck cancer.

### 3.4. Ki67

Ki67 (MKI67) is a non-histone nuclear protein whose gene is located on chromosome 10q25 and encodes two marker isoforms differing in molecular weight [39,40]. Ki67 is a protein distributed in the nucleus and nucleolar cortex. It is involved in the regulation of cell proliferation. Expression of this molecule has been shown to be particularly high in poorly differentiated tumours and may serve as a prognostic biomarker for solid tumours [40]. As regards assessing the rate of cell division using this molecule, it is important to note that its expression takes place only in the interphase of the cell cycle, increasing with its progression, with no expression occurring in the G0 phase. The highest levels of this marker are observed in late S and G2 phases, with a rapid drop occurring immediately after mitosis [41]. Therefore, the assessment of the so-called Ki67 index indicates the rate of tumour growth, making it potentially helpful in determining tumour aggressiveness and prognosis. Hence, it is a valuable prognostic indicator, significantly associated with the risk of death in laryngeal cancer. What is more, it may be helpful in the histological characterisation of glottic squamous cell tumours [39,42]. Furthermore, Ki67 correlates with the stage of SCC and the presence of lymph node metastases, allowing for a more accurate patient assessment and the selection of a well-targeted, aggressive therapeutic approach [43]. A similar prognosis has been described for oral squamous cell carcinomas, where overexpression of Ki67 correlated with poorer prognosis [44]. Most studies correlate Ki67 expression with clinical outcomes, describing this marker as having prognostic, but rarely predictive, value [45]. In general, patients with squamous cell carcinomas of the larynx with limited Ki67 expression had a lower degree of histological differentiation of the tumour, higher sensitivity to treatment, and lower recurrence rate, translating into better five-year-survival compared to the group showing high expression and overexpression of this molecule [42,46]. Accordingly, Ki67 has been recognised as a critical marker of cell proliferation. Its increased expression in many cancers, including those of the larynx, significantly correlates with the rate of cell proliferation, as well as the aggressiveness and invasiveness of the tumour. Assessments of this marker should, therefore, be helpful for prognosis in laryngeal cancer, providing important information on the stage of the disease, its classification, and final prognosis [39].

Studies by Grzanka et al. and Kręcicki et al. suggest that Ki67 expression correlates with the expression of the PCNA in proliferating epithelia of laryngeal cancer and that both proteins may serve as markers of neoplastic growth within this organ [41,47]. PCNA, in addition to Ki67, is, therefore, another important marker involved in the regulation of the cell cycle.

### 3.5. PCNA

PCNA, like Ki67, is expressed in the late G1 phase, reaches its maximum expression in the S phase of the cell cycle, and systematically decreases during the mitotic phase. Trace amounts of this protein are present in the G0 phase. It is a stable, toroidal-shaped homotrimer found in many types of human cancers, correlating with the course of carcinogenesis and the prognosis of oncological patients. The PCNA localises primarily to the cell nucleus, binding to heterochromatin and euchromatin. It regulates DNA polymerase activity there and is involved in DNA replication and new DNA strand formation. The presence of this protein has also been demonstrated in the cytoplasm, indicating its involvement in biosynthetic pathways [41,47,48,49,50].

Positive PCNA staining has been demonstrated in most laryngeal tumours. The expression distribution of this marker, however, is heterogeneous, indicating areas of high and low proliferative activity, meaning a differential distribution of highly and poorly differentiated cells. This supports the assumption that there is a correlation between PCNA expression and the degree of histological differentiation of laryngeal cancers and, consequently, their advancement. Furthermore, PCNA expression levels correlated with the response of oral cancer patients to chemotherapy [51]. This means that the assessment of this protein’s level can help to determine the efficacy of potential treatment and identify patients with favourable prognosis. A correlation was also found between the stage of development of laryngeal tumours and the degree of PCNA marker expression by tumour cells. Unfortunately, a correlation between PCNA expression levels and potential disease recurrence could not be confirmed [51,52]. Other studies indicated the absence of another correlation—between the degree of PCNA expression and the occurrence of metastases to regional lymph nodes in patients with laryngeal cancer [41]. Generally speaking, the expression of both the Ki67 antigen and PCNA can be considered a marker of the proliferation rate of laryngeal epithelial cells, reflecting the progress of carcinogenesis in the larynx [47].

## 4. Structural Regulators

Another group of potential markers in laryngeal cancer can be described as structural regulators. These include, for example, E-cadherin and integrins, CD44, epithelial membrane proteins (EMPs), cortactin (CTTN) or the focal adhesion kinase (FAK).

### 4.1. E-Cadherins and Integrins

E-cadherins are transmembrane glycoprotein adhesion molecules grouped mainly at the site of direct contact between neighbouring epithelial cells. They are also directly connected to the cell cytoskeleton via β-catenins. This enables not only the maintenance of epithelial structure or cellular signal transduction but also the phenotypic modification of epithelial cells, as well as the acquisition of mobility by polymerisation and depolymerisation of actin filaments [53]. This means that changes in the expression of E-cadherins have important prognostic significance for cancer patients. Indeed, it has been shown that a reduction in the level of these molecules on the cell surface destabilises the E-cadherin/β-catenin complex. This increases the aggressiveness of laryngeal tumours and may entail a poor prognosis in this group of patients [53,54]. What is more, the loss of surface E-cadherins is associated with advanced tumour stages, shorter overall survival, and a high likelihood of metastasis [54]. Furthermore, Le et al. showed that a reduction in the expression of these adhesion molecules may also be associated with the presence of lymph node metastasis [55]. This suggests that the decrease in E-cadherins, which is observed in many cancers, including laryngeal cancer, is a potential predictor of lymph node metastasis [56,57]. Nevertheless, there is controversy in the literature regarding the recognition of this particle as an unequivocal marker of clinicopathological features in laryngeal cancer [58,59].

Integrins are heterodimeric transmembrane proteins consisting of α and β subunits. They are receptors that function as cell–cell and cell–matrix communication molecules. One of the molecules belonging to the ITGA group is integrin subunit α 5 (ITGA5) [60]. In most cases, it recognises its specific ligand through α5β1 heterodimer formation. It has been shown to be highly expressed on many cancer cells, including laryngeal cancers, and associated with their malignant phenotype expressed through proliferation, epithelial–mesenchymal transition (EMT), migration, and invasion. This is due to the fact that ITGA5 is involved in the processes of dynamic transformation of the extracellular matrix [61,62]. It has also been shown that ITGA5 not only regulates the malignant phenotype of cancer cells but also influences immune infiltration through ECM remodelling. Liu et al. demonstrated a strong ITGA5-TGFB1-PDCD1LG2/CD47 correlation, which exerts a significant immunosuppressive effect on the tumour microenvironment and may promote the progression of head and neck squamous cell carcinomas (HNSCC). Furthermore, this phenomenon is exacerbated by the association of ITGA5 with the infiltration of immunosuppressive M2 macrophages [61]. Reduced immune cell infiltration correlates not only with ITGA5 but also with ITGA3 and ITGA6. This, in turn, contributes to the development and invasion of head and neck cancers by activating signalling pathways such as FAK and PI3K [62]. Levels of ITGA5 in LSCC are known to be elevated compared to normal tissues. This is associated with shorter overall survival (OS) in this group of cancer patients. ITGA5 overexpression in LSCC can therefore be considered as an independent unfavourable prognostic factor, biomarker, and predictor of prognosis and response to chemotherapy, immunotherapy, or targeted therapy in this group of patients [60,62].

### 4.2. CD44

CD44 is a transmembrane glycoprotein promoting the adhesion of malignant cells to vascular endothelial cells. Furthermore, this molecule is involved in not only cell–cell but also cell–matrix and cellular signalling relationships [63,64]. CD44 has been shown to be frequently dysregulated in cancer cells of various origins (prostate, ovarian, lung, or pancreatic cancers), as well as in laryngeal carcinomas [64]. Head and neck cancers specifically tend to show overexpression of the CD44 molecule compared to other solid tumours. However, in the context of these neoplasms, the specific location of the tumour lesion must be taken into account. This is because literature data indicate that sometimes differential expression of CD44 is associated with the metastasising behaviour of tumour cells. It has been shown that in oral squamous cell carcinoma (OSCC), loss of expression of certain CD44 variants, i.e., a decrease in the amount of the molecule detected, correlates with a higher histological tumour stage and metastatic activity of cells [65,66]. In contrast, Kokko et al. indicated that overexpression of CD44 could be considered a predictor of aggressiveness, including a high metastatic potential of pharyngeal and laryngeal head and neck squamous cell carcinoma (HNSCC) cells [67]. CD44 is also implicated as a potential marker for more aggressive forms of laryngeal carcinomas. Moreover, it is important to consider the co-expression of CD44 with other proteins, as it is also involved in regulating the aggressiveness of laryngeal carcinomas. One such additional protein is autophagy-related protein 7 (ATG7), which plays a protective role against complex disease states. Hence, dysfunctions in the structure or function of ATG7 may be associated with disease symptoms, including cancer development [68]. It is assumed that overexpression of CD44 with concomitant impairment of ATF7 activity may be the basis for expecting a high risk of lymph node metastasis and distant metastasis in laryngeal cancer patients [69]. In addition, attention has been drawn to the association of CD44 and microRNAs (miRNAs), which are endogenous non-coding RNAs, and its significance for laryngeal tumour aggressiveness. miRNAs serve an important function in the body by regulating more than 30% of genes at the posttranscriptional level. This suggests their significant involvement in both physiological and pathological conditions [70]. An important one is miR-373, as it posttranscriptionally regulates large tumour suppressor kinase 2 (LATS2), promoting laryngeal carcinoma metastasis [71]. In addition, miR-373 shows correlation with CD44 and E-cadherin and can be considered a diagnostic or prognostic biomarker in laryngeal carcinomas [64]. Another molecule whose presence correlates with CD44 expression and prognosis for patients with laryngeal carcinoma is osteopontin. It is a cytokine that binds to the CD44v6 receptor on the cell surface. Celetti et al. showed that levels of this cytokine were high in invasive carcinomas of the larynx and correlated with tumour stage, lymph node tumour cell spread, distant metastases, and overall survival. High osteopontin levels also correlated with CD44v6 expression on tumour cells. This implies that osteopontin may be a specific biomarker in aggressive laryngeal squamous cell carcinomas, suggesting poor outcomes for patients with high levels of this cytokine [72]. CD44 is, therefore, an important marker molecule for laryngeal tumours, indicating the presence of a highly aggressive mass that is, presumably, not necessarily related to the stage of the disease.

### 4.3. Cortactin (CTTN)

Cortactin (CTTN) is present in all cell types. It is a ubiquitous multidomain protein found in the cytoplasm. After stimulation, it takes part in actin filament polymerisation or rearrangement. Due to its regulatory role in cytoskeleton organisation, as well as adhesion molecule intracellular signalling and extracellular matrix (ECM) modulation, cortactin may be considered an important factor related to cell mobility and cancer metastasis [73]. CTTN locus is in the 11q13 region, which is known to be amplified in many human cancers, including those of the larynx. The amplification of this region is a common finding in laryngeal tumours. What is more, it is associated with distant and lymph node metastases, predicting poor prognosis for the patient [74]. The amplification of this region and CTTN protein overexpression are also associated with the overactivation of the epidermal growth factor receptor (EGFR)-ERK signalling pathway and increased levels of EGFR on tumour cells. This results in resistance to cytostatics, ionising radiation, and ultimately, further disease progression [73,75]. Furthermore, the poor prognosis of patients with CTTN overexpression is associated with a concomitant increase in the level of cyclin D1. Encoded in the 11q13.3 region, it is a proto-oncogene that regulates the cell cycle and exhibits high expression in many tumours. However, in the case of laryngeal cancer, cyclin D1 level is not a sufficient marker because the prevalence of its overexpression in these tumours is so high that it alone does not constitute a clear prognostic or predictive factor in this group of patients. However, since cortactin is a significant gene in the 11q13.3 amplicon, both proteins may be considered predictors of high mortality resulting from the spread of laryngeal carcinoma cells, especially in advanced stages [74,75]. It should be noted, however, that cortactin may influence the overall aggressiveness of head and neck cancers regardless of the degree of 11q13 amplification. An important parameter that could serve as a grading system in laryngeal carcinoma patients may be the expression of CTTN in the superficial and deep invasive front of the cancer. Differences may be related to the prognosis resulting from the likelihood of metastasis, but this should not be associated with the initiation of carcinogenesis [75].

### 4.4. Focal Adhesion Kinase (FAK)

Amplification of the chromosomal region 8q23-24 is also frequently observed in head and neck cancers. It is even believed to be an early chromosomal event in head and neck carcinoma progression. This region contains, among others, FAK/PTK2 genes, which encode focal adhesion kinase (FAK). FAK is an intracellular tyrosine kinase protein activated in response to integrin clustering [76]. This factor is also referred to as a survival signal for anchorage-independent cells because it plays an important role in the control of cell-extracellular matrix interactions. Consequently, a reduction in the expression of this factor will be associated with an increase in apoptosis of tumour cells. By extension, the overexpression of FAK observed in these cells can be seen as a defence mechanism to avoid anoikis not only during infiltration of surrounding normal tissues, but also movement within the vascular beds. Accordingly, it has been suggested that the aberrant expression of this kinase may be important in the acquisition of malignant features by the cells, manifested particularly by the increased mobility potential of these cells and the presence of nodal metastasis. This is the case, among others, in laryngeal carcinomas, in which the overexpression of this molecule is considered a predictor of lymph node metastasis and poor prognosis [77,78]. Furthermore, the co-occurrence of FAK and CTTN overexpression in dysplastic lesions of laryngeal cancer may suggest the possibility of a significant, high-risk malignant lesion within a relatively short period of time. It has also been suggested that expression levels of these markers may provide a stronger predictive signal than the standard histological grading [79]. Focal adhesion kinase (FAK) thus represents an interesting form of marker with proven applicability in laryngeal cancer.

### 4.5. Epithelial Membrane Proteins (EMPs)

Epithelial membrane proteins (EMPs) are an interesting set of factors strongly associated with the development of many types of cancer. They belong to the peripheral myelin protein (PMP22) gene family. The proteins encoded by this gene family are responsible for the regulation of cell growth and cell proliferation, including tumour development and metastasis [80]. There are three basic types of EMPs (EMP1, EMP2, EMP3). However, it has been indicated that depending on the type of tumour cells and their tissue origin, EMPs may have different functions, and their variable expression may cause a range of phenomena in pathological tissue [81]. EMP1 acts through the PI3K/AKT pathway to regulate cell adhesion, EMP2 acts through the FAK/SRC pathway to regulate cell migration, and EMP3 acts through the ErbB2-PI3K-AKT pathway to affect proliferation, differentiation, apoptosis, survival, and metastasis [82]. Due to the different molecular pathways of EMP-mediated signal transduction and the expression levels of these proteins, both suppressive and progressive effects of this signalling on cellular processes are observed, depending on the type of cancer [83]. Head and neck cancers show high expression levels of EMP1 and EMP2 forms, while nasopharyngeal cancers exhibit high protein levels of EMP2 and EMP3. Conversely, low expression levels of EMP family proteins have been found in oral cavity and nasopharyngeal cancers [82]. Other studies indicate that laryngeal, oral, and nasopharyngeal carcinomas express reduced levels of EMP1 [84,85]. However, EMP family proteins are not prevalent as markers, but rather as possible therapeutic targets. Particularly notable in this context is EMP3, which acts as a tumour suppressor protein in some cancers and affects the mobility of oral squamous cancer cells along the miR-765-EMP3-p66Shc axis [86]. This implicates it as a marker of proliferation and cell mobility in the cancer, as well as a potential therapeutic target. Based on the available literature, scientific evidence remains insufficient to clearly define EMPs as fully reliable biomarkers. Further research and clear validation are needed to verify their usefulness. Nonetheless, under current evidence, they could be considered a potential therapeutic target in the treatment of laryngeal cancer.

## 5. Immune Factors

### 5.1. Programmed Cell Death Ligand (PD-L1)

Among immune-mediated factors, the PD-1 receptor ligand (PD-L1) is often taken into consideration for laryngeal carcinoma analyses. The programmed death protein 1 (PD-1) and its programmed cell death ligand (PD-L1) are among the important checkpoint molecules. Their interaction inhibits T-lymphocyte activity while enhancing the Treg-associated response. This involves maintaining homeostasis within tissues at the level of immunological phenomena. Tumour cells use this system to escape the regulatory activity of the immune system. Hence, the idea of limiting the functionality of the PD-1/PD-L1 pathway in tumour tissue and re-establishing full T-cell activity appears to be valid, as it enhances the anti-cancer response of the patient’s immune system and represents an interesting alternative in immunotherapy [87]. Analyses of the PD-L1 ligand, however, point to its indeterminate role as a potential marker of laryngeal tumour growth processes. In addition, attempts to estimate patient prognosis on the basis of its quantity may not seem warranted at present. Three conclusions can be drawn from these analyses. First, this factor may be applicable as a potential marker. The ligand may serve as an indirect marker of how effectively laryngeal squamous cell carcinoma (LSCC) responds to treatment. According to the authors, this is mainly expressed through the increased activity of the immune system against tumour cells [88]. Franz et al. indicated that in relation to the combined positive score, PD-L1 may be associated with longer disease-free survival and a reduced risk of recurrence in laryngeal carcinomas [89]. It is also indicated that gene expression and expression of the PD-1 receptor and its ligand PD-L1 are significantly elevated in laryngeal carcinomas. Moreover, the higher the expression of these factors, the more advanced the tumour. It follows that a higher expression of PD-1/PD-L1 both on tumour cells and in their local microenvironment should be associated with a better tissue response to immunotherapy. In these terms, these molecules have been recognised as a useful prognostic marker in laryngeal carcinoma patients [90]. Studies by Molga-Magusiak et al. have suggested that the PD-L1 ligand could be useful for the prognosis of malignant laryngeal lesions. They observed that the soluble form of this ligand, sPD-L1, may serve as a predictive marker to help evaluate malignant lesions located in the head and neck region. The studies also indicated a promising role for this ligand in the early detection of malignant laryngeal lesions. According to the authors, sPD-L1 could hence serve as both a prognostic and predictive serum marker for head and neck tumours [91]. The second conclusion is that the PD-1 receptor ligand is significant but in conjunction with other factors. Assessment of PD-L1 and selected tumour microenvironment factors, e.g., tumour-infiltrating lymphocytes (TIL) count or tertiary lymphoid structures (TLS), may have relevance in patients at a higher risk of laryngeal carcinoma recurrence [92]. Similarly, CD103+ TILs correlated positively—though inconspicuously—with PD-L1 in laryngeal carcinoma [93]. Also, negative PD-L1 expression with a high CD8+/FOXP3+ ratio may serve as a marker (biomarker) in patients with squamous cell carcinoma of the larynx (SCC-L) [94]. It has also been shown that the coupled analysis of PD-1, PD-L1, and apurinic/apyrimidinic endonuclease 1 (APE1) can be used as a biomarker providing predictive value for laryngeal and hypolaryngeal carcinomas. This will enable the appropriate selection of checkpoint inhibitor-based treatment in the group of patients most likely to benefit from this treatment modality [95]. The third conclusion is that the PD-1/PD-L1 pathway does not have a significant marker potential in laryngeal carcinoma patients. The authors of the studies indicated that PD-1, PD-L1, and PD-L2 could not be associated with prognosis for LSCC. Furthermore, there was no correlation between PD-L1 expression and overall survival in head and neck squamous cell carcinomas (HNSCC). Other studies have also failed to demonstrate significance in PD-L1 expression and the number of TILsCD8+ and thus prognostic value in a group of patients with laryngeal carcinoma receiving postoperative radiotherapy (PORT) [96,97,98]. These ambiguities warrant further studies, perhaps with a focus on patients with head and neck cancer. These patients may derive measurable therapeutic benefits from the evaluation of PD-1/PD-L1 pathway expression.

Differences in the described PD-L1 expression may result from various factors. Biological factors are of primary significance, but the test results also depend on technical conditions and even sample collection methods. Biological factors associated with differential PD-L1 expression can be classified as genetic changes in the analysed cells, selectively amplifying genes for this factor or affecting its structure, or the activity of the tumour microenvironment (TME) related to hypoxia or acidification, which regulates certain signalling pathways in tumour cells. Furthermore, the varied and sometimes distinct PD-L1 expression may be influenced by tumour characteristics resulting from acquired distinct genetic profiles among tumours classified into the same type or their histological differences. Furthermore, PD-L1 levels can be determined by analyses of different stages of specific tumours, with material isolated from the primary tumour or metastatic sites. This is due to the fact that PD-L1 is a heterogeneous molecule, exhibiting dynamic variability across tumour regions. Technical considerations include differences in the applied tests, the types of antibodies used, the potential level of sample degradation, prior patient treatment, and observational variability related to the interpretation of the results by a pathologist or researcher. Therefore, PD-L1 levels are not a perfect marker, requiring very careful analysis and interpretation of the obtained data. It is important to note, however, that even patients with low or undetectable PD-L1 levels can derive measurable benefits from anti-PD-1/PD-L1 therapy.

### 5.2. Immune Cells

It has been suggested that the levels of specific immune cells, as well as their mutual proportions and relationships, have a significant impact on the development and progression of laryngeal tumours. This means they could be useful for staging, determining patient risk, or making treatment decisions. Serum markers relevant to the development of local inflammation, such as the neutrophil-to-lymphocyte ratio (NLR), platelet-to-lymphocyte ratio (PLR), and lymphocyte-to-monocyte ratio (LMR), can be considered and analysed to potentially predict tumour prognosis, correlating the inflammatory response and the occurrence of tumour cell resistance to treatment [99].

LMR is closely associated with tumour recurrence and the presence of lymph node metastases and thus can be linked to prognosis. It is examined in terms of the patient’s immune system balance and the development of the tumour microenvironment. Elevated NLR levels correlated with overall survival (OS) but appeared to be unrelated to local recurrence-free survival (RFS) in patients with squamous cell carcinoma of the larynx [100]. Zhao et al., however, indicated that elevated NLR levels were associated with poor prognosis and could be considered a potential biomarker of poor prognosis in patients with LSCC [99]. Elevated PLR levels in laryngeal cancer patients before treatment indicated a poorer prognosis. Therefore, after further extensive research, they could be considered a potential biomarker predicting prognosis in this group of patients [101]. Furthermore, additional immunological parameters, such as the ratio of cytotoxic T-lymphocytes to immunosuppressive cells (CIL) and the level of tumour-infiltrating lymphocytes (TIL), were studied in the context of predicting survival outcomes in patients with laryngeal carcinoma. Research by Zhang et al. indicated that CIL could be considered a marker with prognostic value in LSCC and could thus be helpful in making treatment decisions in this group of patients [102]. Finally, a high TIL index is associated with higher survival rates and, consequently, a better prognosis for patients with HNSCC [103].

The values of these parameters are closely related to the levels of specific cells, such as anti-inflammatory (N1) and pro-inflammatory (N2) neutrophils and differentiated T lymphocyte subpopulations (CD8+, CD3+, or Treg). Their potential is associated with the intensity of inflammation and regulation of antitumour immunity, as well as with determining tumour cell mobility and the occurrence of metastases. However, due to the lack of sufficient validation, interrelationships between immune cells are not widely used in practical diagnostics; however, they may serve as auxiliary biomarkers in screening analyses and for predicting the prognosis of patients with laryngeal carcinoma.

## 6. Molecules Involved in Growth Factor Pathways

Transforming growth factor-β (TGF-β) and epidermal growth factor (EGF) are remarkable in that they represent molecules involved in growth factor pathways and, under the right conditions, can be treated as markers.

### 6.1. Transforming Growth Factor β (TGF-β)

Transforming growth factor β (TGF-β) has been shown to either stimulate tumour growth (advanced stages) or inhibit—or, more specifically, limit—tumour growth (early stages). The levels of mRNA and protein expression for TGF-β1 and -β2 are elevated in tumour tissue compared to normal tissue. This implies the potential of using mRNA and protein levels for this factor as a marker to distinguish laryngeal carcinoma from surrounding normal tissue [104]. Logullo et al. came to the opposite conclusion. They found that the cells of this tumour produced less TGF-β1 than surrounding normal cells. A loss of production of this factor would be an ordinary, typical phenomenon in cells originating in the head and neck region. Furthermore, the variability of TGF-β1 expression in this type of tumour essentially disqualifies it as a marker that can inform the prognosis of laryngeal cancer patients. Accordingly, the authors concluded that TGF-β1 did not correlate with clinicopathological parameters, making it difficult to establish a clear link between its expression and disease progression [105]. Nevertheless, TGF-β1 is the predominant form of this factor in tumour tissue, significantly influencing the development of local inflammation, driving tumour cell proliferation and, as a result, potentially affecting tumour cell behaviour. The conflicting results may be due to the different expression of this factor in tumour tissues from different anatomical locations of the head and neck region. Furthermore, they may show equal sensitivity to high or low local concentrations of this molecule. And finally, it is important to consider the stage of the tumour where TGF-β1 is activated or remains in a latent state, depending on the acidification of the local microenvironment. Published data indicate that the high variability of TGF-β1 levels due to multiple environmental factors limits its usefulness as an independent tumour biomarker in laryngeal cancer.

### 6.2. Epidermal Growth Factor (EGF)

The second factor mentioned is epidermal growth factor (EGF). According to a study by Goel et al., the receptor for this factor (EGFR) may serve as a prognostic marker in laryngeal squamous cell carcinomas [106]. Other studies point to epidermal growth factor-like domain 7 protein as a factor whose overexpression in laryngeal squamous cell carcinoma may be associated with lymph node metastasis and distant metastasis, translating into poor prognosis for the patient. It could, therefore, be considered a potential prognostic marker in this cancer [107]. A correlation between EGF receptor (EGFR) levels and patient prognosis was also demonstrated by Marijic et al. They showed that after tissue stimulation, the membrane form of EGFR (mEGFR) is displaced into the nucleus, and the expression and localisation of such a nuclear form of the receptor could be associated with poor prognosis for laryngeal carcinoma patients [108]. However, as was the case with VEGF, EGFR should also be developed together with COX-2 expression. EGF stimulates COX-2 expression and activity in tumour cells and consequently contributes to the metastatic nature of pathological tissue [109].

## 7. Hormone Receptors

The pathogenesis of laryngeal carcinomas also implicates hormone receptors as potential developmental markers for these tumours, as well as indicators of lymph node metastasis and distant metastasis. Such analyses are warranted, as up to 70% of all tumour types categorised as laryngeal carcinomas may express oestrogen receptors (ERs) and respond to the presence of 17β-oestradiol in the tumour environment [110]. However, as with PD-1/PD-L1, there are discrepancies regarding the importance of this receptor in patient prognosis resulting from tumour invasiveness. They exist at two levels. The first relates to differences in the interpretation of tumour invasiveness assessment based on the expression of specific receptors found. The second generally relates to the inability to identify the presence of such receptors on tumour cells and thus the lack of validity of their evaluation as potential markers in laryngeal carcinoma. Verma et al. indicated that the loss of ERα as well as ERβ, in contrast to ERα36, was associated with an increase in the aggressiveness of LSCC, possibly expressed by advanced stage clinicopathological assessment or increased lymph node metastases. The site of ER expression was also important, as changes in the expression of nuclear—rather than membrane—forms were responsible for this effect [110]. Another interpretation suggests that high ERβ expression limits the acquisition of an aggressive phenotype by tumour cells, expressed by the stimulation of the epithelial–mesenchymal transition (EMT) process and consequently a decrease in E-cadherins and induction of cell mobility. This would warrant the use of the receptor as a marker in early stages of laryngeal carcinoma, where such an effect has been observed [111]. Furthermore, high expression of this receptor was associated with survival of patients with oropharyngeal cancer [112]. Conversely, some studies noted that high expression of ERβ and progesterone receptors (PR) and low expression of androgen receptors (AR) characterised poorly differentiated laryngeal carcinomas showing a tendency towards lymphatic metastasis. Similar observations were made by Fei et al., indicating higher mRNA and protein expression of AR, ERα, and prolactin receptor (PRLR) in LSCC than in adjacent normal tissues [113]. In this case, the expression of these receptors can also be considered a marker, albeit one of poor prognosis and aggressiveness of the tumour. These receptors can also be considered here as potential targets for anti-receptor therapy [114]. There are also reports in the literature indicating that the said receptors cannot be identified on laryngeal carcinoma cells. This would indicate a lack of hormone dependence in the process of carcinogenesis, suggesting that the receptors are of no use as potential markers in this cancer [115].

## 8. Factors Regulating Angiogenesis

These factors can also provide an indication of the advancement of laryngeal carcinoma. A crucial element in overall tumour progression, microvascular density (MVD) can also be used as a prognostic factor in laryngeal cancers. This is due to the demonstrated correlations linking the density of newly formed blood vessels with disease progression and with shorter overall and disease-free survival rates [1]. Many factors related to vascularisation have been studied for their potential role as specific markers. The molecules studied include CD105, CD31, epidermal growth factor receptor (EGFR), vascular endothelial growth factor (VEGF) and its receptor VEGFR, and angiogenin [116].

### 8.1. Endoglin (CD105)

Endoglin (CD105) is a disulphide-linked, proliferation-associated, hypoxia-inducible homodimeric glycoprotein, being a cell-surface co-receptor of transforming growth factor β (TGF-β) [56,117]. Endoglin is expressed on endothelial cells of newly formed vessels, and, in turn, panendothelial antigens are expressed on both newly formed and pre-existing vessels. In addition, CD105 antibodies were more specific to the vascularisation of tumour lesions [118,119]. By extension, the immunohistochemical staining of the CD105 molecule may be considered a prognostic marker in laryngeal cancer. Moreover, it may be interesting to combine CD105 analysis with VE-cadherin or CD31 molecule expression to improve clinical assessment and survival estimation in laryngeal cancer patients [56].

### 8.2. CD31 and VEGF

CD31 belongs to the immunoglobulin superfamily adhesion molecule and is involved in the progression of vascularisation, being a marker of MVD in tissues of many cancers. Together with CD105, it may be a predictor of recurrence in laryngeal cancer [120]. Increased recurrence and poor prognosis are also associated with high CD31 and VEGF expression in tumour tissue. However, Schulter et al. indicated that, in principle, both CD31 and VEGF may be useful as biomarkers of tumour vascularisation only in patients with early-stage laryngeal cancer [121]. VEGF stimulates tumour cells in situ, which emerge from the quiescent state and, penetrating the basement membrane barrier, begin the process of infiltration and subsequent metastasis. Hence, the expression of this factor in tumours, including laryngeal cancer, may closely correlate with the presence of cancer cells in lymph nodes and, by extension, poor prognosis and survival of laryngeal carcinoma patients [122]. It is estimated that the VEGF-C form is particularly highly expressed in laryngeal carcinoma, which affects the overall clinicopathological assessment of patient prognosis [123]. Furthermore, it has been shown that a homozygous genotype (GG) of the VEGF gene may be a risk factor for laryngeal carcinoma. Thus, the polymorphism of the VEGF gene may provide a basis for its consideration as a potential genetic marker for the development and malignancy of laryngeal SCC [124].

### 8.3. COX-2

The interplay between VEGF and cyclooxygenase-2 (COX-2) is also important in the growth and invasive nature of laryngeal carcinoma. Prostaglandins produced with COX-2 modulate VEGF levels and activate endothelial cell proliferation, contributing to the progression of angiogenesis in pathological tissue. The correlation of pro-cancerous COX-2 and VEGF activity is particularly more pronounced in advanced stages of laryngeal carcinoma than in early stages. This is one of the reasons for viewing this correlation as influencing the invasive and metastatic properties of this tumour [125]. A different interpretation was proposed by Ranelletti et al. According to it, COX-2 is overexpressed in low stages of laryngeal carcinoma, while in higher stages—which show a high malignant potential—the expression of this factor decreases significantly [109]. Xu et al. suggested that microRNA-203 (miR-203) may function as a suppressor of this type of tumour development by downregulating VEGF expression and reducing tumour cell proliferation and mobility [126]. There are conflicting reports in the literature regarding the usefulness of this factor as a potential biomarker in laryngeal carcinomas. These may be due to many factors, but the most important are biological ones, including variations in the tumour itself, analyses of different tumour stages, microenvironmental influences (TME), analysis of material from different tumour locations, or description of COX-2 levels in tissues from primary tumours or metastases. Due to the discrepancies in interpretation, this molecule should be evaluated with great caution as a potential marker of the neoplastic process in laryngeal carcinomas.

### 8.4. Angiogenin

Angiogenin is another factor regulating angiogenesis. It belongs to the ribonuclease superfamily. It has been shown to regulate the vascularisation process in a number of activities, including ribonucleolytic activity, as well as activities modulating the structure of the basement membrane and regulating the transmission of molecular signals [127]. Here, it is also important to consider processes of reciprocal regulation within normal as well as cancerous tissues. Research in this area was conducted by Lovato et al., who evaluated potential relationships between proangiogenic angiogenin and anti-angiogenic tumour suppressor Maspin (mammary serine protease inhibitor) in tumour tissues isolated from the larynx. Elevated levels of angiogenin were shown to correlate with a non-nuclear pattern of Maspin expression in laryngeal cancer. In turn, elevated levels of this inhibitor have been associated with regional recurrence and shorter survival [128]. Angiogenin and Maspin may, therefore, be interesting markers in laryngeal cancer, but in a reciprocal context that seems to better describe the degree of tumour progression and prognosis than assessing these parameters individually.

## 9. Conclusions

In summary, many of the factors discussed here are being investigated for their usefulness as biomarkers in laryngeal cancer. These can serve as markers indicating the presence and progression of pathological tissue growth. Such proteins include cell cycle regulators; apoptosis regulatory proteins; products of oncogenesis and tumour suppressor genes; molecules involved in growth factor pathways; angiogenic, structural, or immunological factors; and sex hormones. However, many of the factors belonging to these groups cannot always be considered independent, stand-alone biomarkers. Some of them should be developed in conjunction with other molecules, as this can significantly improve their effectiveness. Another crucial element in assessing marker expression is their subcellular location. The accurate identification of the cellular compartment where a specific factor is overexpressed can only provide insights into the characteristics of the tumour. Often, the expression of a specific protein is classically so high that it alone cannot provide a clear prognostic or predictive indication in patients with laryngeal cancer. It is also important to pay attention to literature reports, as some markers may only demonstrate predictive or prognostic value. Some biomarkers can also serve as both an effective indicator of the presence or progression of cancer and a potential therapeutic target. Targeting these proteins may be a good alternative to standard therapeutic regimens. Some tumour markers may provide a viable alternative to traditional methods based on histological grading, as tumour aggressiveness does not necessarily correlate with disease progression. Hence, by analysing selected markers, it is possible to improve the assessment of patient prognosis regardless of the tumour stage. Furthermore, many markers can effectively indicate the potential presence of metastases, which not only disseminate through the bloodstream but also tend to penetrate the lymphatic system. Moreover, biomarkers may indicate the chemosensitivity of certain head and neck cancers. Their assessment could allow for more effective selection of chemotherapy treatments to reduce potential side effects.

Nevertheless, there are reports in the literature suggesting that certain proteins cannot be considered unequivocal markers of clinicopathological features in laryngeal cancer. Some proteins considered potential markers of laryngeal cancer are characterised by relatively high expression variability. Their expression depends on too many variables to be considered a factor in making or supplementing prognosis. This may be due to the characteristics of the marker itself, the stage of the tumour, its location, structure, etc. The selection and diagnostic efficacy of biomarkers in laryngeal cancer can hence be influenced by multiple variables. Many factors meet the scientific criteria for being recognised as cancer biomarkers. Unfortunately, for various reasons, they lack clinical utility. In this paper, we focused on a general presentation of factors that have been or are being analysed as potential biomarkers in laryngeal cancer without defining their usefulness in absolute terms.

## Figures and Tables

**Table 1:** List of selected molecules described in this paper as potential tumour markers in laryngeal cancer.

Group of Factors	Molecule Analysed as a Potential Marker
Genetic and apoptosis-associated factors	1. Long non-coding RNAs (IncRNAs) 2. Proteins related to apoptosis
Cell cycle regulators	1. Cyclin D1 2. p16 protein 3. p27 protein 3. Ki67 5. PCNA
Structural regulators	1. E-cadherins and integrins 2. CD44 3. Cortactin (CTTN) 4. Focal adhesion kinase (FAK)
Immune factors	1. Programmed cell death ligand (PD-L1) 2. Immune cells
Molecules involved in growth factor pathways	1. Transforming growth factor β (TGF-β) 2. Epidermal growth factor (EGF)
Hormone receptors	1. Oestrogen receptors (ERs) 2. Progesterone receptors (PR) 3. Prolactin receptor (PRLP) 4. Androgen receptors (AR)
Factors regulating angiogenesis	1. Endoglin (CD105) 2. CD31 and VEGF 3. COX-2 4. Angiogenin

## Data Availability

No new data were created or analyzed in this study, data sharing is not applicable.
